# The Core Complex of the Ca^2+^-Triggered Presynaptic Fusion Machinery

**DOI:** 10.1016/j.jmb.2022.167853

**Published:** 2022-10-13

**Authors:** Axel T. Brunger, Jeremy Leitz

**Affiliations:** Department of Molecular and Cellular Physiology, Stanford University, Stanford, United States; Department of Neurology and Neurological Sciences, Stanford University, Stanford, United States; Department of Structural Biology, Stanford University, Stanford, United States; Department of Photon Science, Stanford University, Stanford, United States; Howard Hughes Medical Institute, Stanford University, Stanford, United States

**Keywords:** neurotransmitter release, synaptic vesicle fusion, cryo-electron tomography, stapled peptide, mucin secretion

## Abstract

Synaptic neurotransmitter release is mediated by an orchestra of presynaptic proteins that precisely control and trigger fusion between synaptic vesicles and the neuron terminal at the active zone upon the arrival of an action potential. Critical to this process are the neuronal SNAREs (Soluble *N*-ethylmaleimide sensitive factor Attachment protein REceptor), the Ca^2+^-sensor synaptotagmin, the activator/regulator complexin, and other factors. Here, we review the interactions between the SNARE complex and synaptotagmin, with focus on the so-called primary interface between synaptotagmin and the SNARE complex that has been validated in terms of its physiological relevance. We discuss several other but less validated interfaces as well, including the so-called tripartite interface, and we discuss the pros and cons for these possible alternative interfaces. We also present new molecular dynamics simulations of the tripartite interface and new data of an inhibitor of the primary interface in a reconstituted system of synaptic vesicle fusion.

## Introduction

Synaptic transmission between pre- and postsynaptic neurons occurs when the presynaptic neuron terminal is temporarily depolarized upon an action potential, opening voltage-gated Ca^2+^ channels at or near the active zones of synapses. Because the extracellular Ca^2+^ concentration is much higher than the basal cytoplasmic concentration, Ca^2+^ will flow into the cytoplasm. In turn, Ca^2+^ will trigger fusion of neurotransmitter-filled synaptic vesicles with the presynaptic membrane in less than a millisecond.^1,2^ Upon fusion, neurotransmitter molecules are released into the synaptic cleft, and then bind to receptors that are in the postsynaptic membrane.

**Figure 1.**
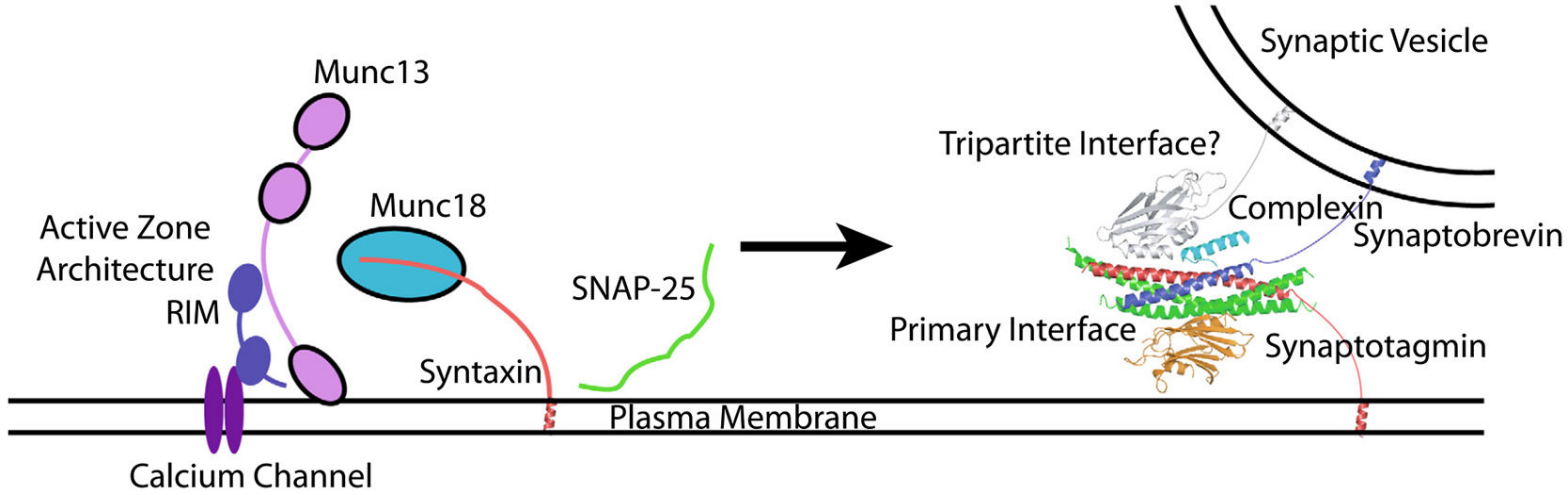
Model of the presynaptic neurotransmitter release machinery. Shown are major active zone proteins (RIMs, Munc13, Munc18, P/Q- or N-type Ca^2+^-channels, SNAREs (SNAP-25, syntaxin, and synaptobrevin), complexin, and synaptotagmin.^91^ On the right side of the drawing, the crystal structure of the SNARE–Cpx1–Syt1 complex is shown^4^ (PDB ID 5W5C), indicating both the primary and tripartite interfaces.

The synaptic vesicle fusion machinery includes the neuronal SNAREs (Soluble *N*-ethylmaleimide sensitive factor Attachment protein REceptor), the Ca^2+^-sensor synaptotagmin, the activator/regulator complexin, the assembly factors Munc18 (mammalian uncoordinated-18), and Munc13 (mammalian uncoordinated-13) (Figure 1). However, the molecular mechanisms of Ca^2+^-triggering, regulation, and membrane fusion are still unclear. Central to these questions is the role of synaptotagmin and how it interacts with the SNARE complex to mediate precisely controlled synaptic vesicle fusion on a submillisecond timescale.

In this article, we review the interactions between the SNARE complex and synaptotagmin that have been uncovered by recent structural/biophysical studies.^3-6^ A major focus is the so-called primary interface between synaptotagmin and the SNARE complex (Figure 1), which has been validated in terms of its physiological relevance by several experiments in neurons. We then discuss several other less validated interfaces as well, in particular the so-called tripartite interface (Figure 1) and discuss arguments for and against these alternative interfaces. We also present new molecular dynamics simulations of the tripartite interface and new data with a reconstituted system of synaptic vesicle fusion for an inhibitor of the primary interface (referred to as SP9).

## SNAREs

In the following, we briefly summarize some of the key findings about neuronal SNAREs – for a recent comprehensive reviews, see refs. 7,8. Briefly, prior to membrane fusion, synaptobrevin-2 (also called VAMP2 – Vesicle Associated Membrane Protein 2) on the synaptic vesicle, and syntaxin-1A and Synaptosomal-Associated Protein, 25kDa (SNAP-25) on the plasma membrane initially form a *trans* SNARE complex, with the transmembrane domains of synaptobrevin-2 and syntaxin-1A in the synaptic and plasma membranes, respectively. During fusion, the SNARE complex completely zippers into the fully assembled *cis* SNARE complex where the soluble core consists of a parallel, four α-helix bundle.^9^

The assembly of the SNARE complex is thought to provide the energy necessary for membrane fusion.^9,10^ Single-molecule optical and magnetic trap pulling experiments suggest that the free energy that is released by the zippering of one SNARE complex is approximately 36 *k*_*B*_*T*.^11,12^ This estimated free energy is somewhat less than the energy that is required to overcome the hydration-force barrier for the formation of a lipid stalk^13^; however, this should be viewed only as a qualitative comparison because the exact free energies depend on the particular membrane composition and molecular environment. In this context, we note that at least two synaptobrevin-2 molecules, and presumably-two SNARE complexes, are required for fast Ca^2+^-triggered exocytosis.^14^

## Synaptotagmins

In the following, we briefly summarize some of the key molecular and functional properties of synaptotagmins (Syts) – for comprehensive recent reviews, see refs.^7,8^. Briefly, Syts constitute an evolutionary conserved family of proteins^15^ that are composed of an N-terminal single transmembrane-spanning domain, a variable juxtamembrane linker, and two C-terminal cytoplasmic Ca^2+^-binding C2 domains, termed C2A and C2B, respectively.^16^ Syts differ in their cellular expression and subcellular localization patterns^17^ in addition to the fusion kinetics they endow. Several Syt isoforms (Syt1, Syt2, Syt9) reside on the synaptic vesicles and are vital for synchronous Ca^2+^-triggered synaptic vesicle fusion^18-20^; these isoforms are strictly coupled to an action potential. In contrast, Syt7 mediates asynchronous release,^21,22^ and it is loosely coupled to an action potential.

Syt1 interacts with anionic membranes and SNARE complexes in both Ca^2+^-dependent and Ca^2+^-independent manners.^3,5,6,19,23-32^ Syt1 function and membrane binding is specific to Ca^2+^ binding to the C2 domains: Mg^2+^ does not trigger synaptic vesicle fusion,^33^ while other divalent cations such Sr^2+^ or lanthanides trigger limited synaptic vesicle fusion with significantly different kinetics and possibly either through Syt-independent or through SNARE-independent mechanisms.^34-37^

SNAREs and Syt1 alone are sufficient to promote Ca^2+^-triggered proteoliposome lipid mixing^38^ and full fusion.^39,40^ However, Ca^2+^-triggered fusion with this minimal system is relatively inefficient. More complete reconstitutions greatly increase the efficiency and synchrony of Ca^2+^-triggered fusion.^41,42^ Syt1 has been implied as an activating factor upon Ca^2+^-binding, for example, by bending membranes^3,43-45^ or bridging membranes.^46-48^ However, such an activating role does not explain the effect of certain dominant negative mutants of Syt1 that abolish evoked release in the background of endogenous wildtype Syt1.^49-51^ Moreover, genetic deletion of Syt increased the frequency of spontaneous release in Drosophila,^52,53^ and a similar phenotype was observed upon deletion of Syt1 in mouse neurons.^54^ Additionally, expression of a dominant negative Syt1 mutant also increased spontaneous release in mouse neurons in a Ca^2+^-dependent fashion,^4^ suggesting that Syt1 also has inhibiting roles at resting Ca^2+^ concentration and that a Ca^2+^-sensor other than Syt1 is important for spontaneous release.

## Complexin

In the following, we briefly summarize some of the key findings about complexin (Cpx, we focus here primarily on complexin-1, Cpx1) – for comprehensive recent reviews, see refs. ^7,8^. Briefly, Cpx consists of four domains: The N-terminal domain is important for activation of synchronous Ca^2+^-triggered release in murine neurons^55-58^; the accessory domain regulates evoked and spontaneous release^55,57^; the central domain is required for all functions of Cpx1 and it binds with high affinity (~10 nM) to the neuronal ternary SNARE complex^59-61^; and the C-terminal domain is involved in vesicle priming and binds to anionic membranes in a curvature sensitive fashion.^57,59,62-64^

In conjunction with neuronal SNAREs and Syt1, Cpx1 increases the Ca^2+^-triggered amplitude and synchrony of proteoliposome fusion, and it also suppresses Ca^2+^-independent single vesicle fusion (content mixing).^65^ By varying the Cpx1 concentration, these single-vesicle studies suggest that the regulatory effect on Ca^2+^-independent fusion and the facilitating role on Ca^2+^-triggered fusion are governed by distinct molecular mechanisms involving different subsets of the four domains of Cpx1. Similarly, genetic experiments in *Drosophila* also suggest distinct mechanisms for activation of fast synchronous release and regulation of spontaneous release.^66,67^

## SNARE–Cpx1–Syt1 prefusion interfaces

Because SNAREs alone mediate constitutive fusion, and Syts are the Ca^2+^-sensor for triggered fusion, these molecules cooperate to reduce or prevent Ca^2+^-independent fusion, and they provide the framework for fast triggered fusion. The molecular basis of this cooperation has been elusive until relatively recently. In the following, in order of validated physiological relevance, we summarize the interactions that have been characterized at the atomic level.

### Primary interface

**Figure 2.**
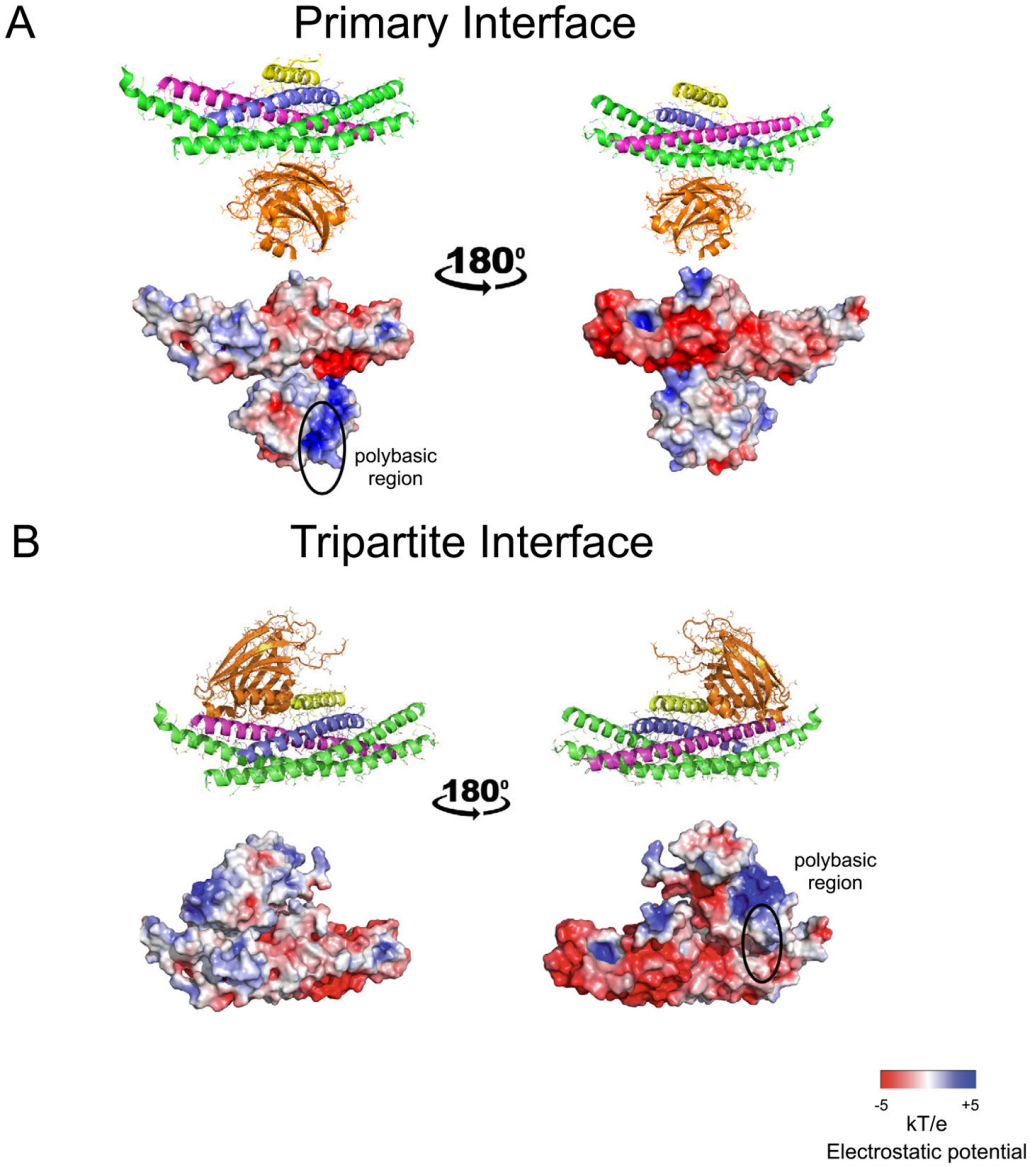
Crystal structures, and charge distributions of primary and tripartite interfaces. (A) The Syt1 C2B domain (orange) is shown that forms the primary interface with the SNARE complex (blue, magenta, green) in the crystal structure of the SNARE–Cpx1–Syt1 complex^4^ (PDB ID 5W5C). The central α-helix of Cpx1 (yellow) is also shown. (B) The Syt1 C2B domain (orange) is shown that forms the tripartite interface with the SNARE complex (blue, magenta, green) and Cpx1 (yellow)^4^ (PDB ID 5W5C). Two views related by a 180° rotation are shown. The top panels are cartoon and bond representations; the bottom panels are electrostatic potential maps. The electrostatic potential maps were calculated with the APBS plugin and displayed using PyMol (Schrödinger, LLC.).

The so-called primary interface between the SNARE complex and the Syt1 C2B domain was first discovered by X-ray crystallography at atomic resolution^3^ (Figure 2(A)). This interface exists both in the presence and absence of Cpx1, and in the presence or absence of Ca^2+^ or Mg^2+^ (PDB IDs 5W5C, 5W5D, 5CCG, 5CCH, 5CCI).^3,4^ For the first structures of this SNARE–Syt1 complex (PDB IDs 5CCG, 5CCH, 5CCI), covalently linked chimeras of the components of the complex were used.^3^ Subsequently, the inclusion of Cpx1 and truncation of the 23 C-terminal residues of the cytoplasmic domain of synaptobrevin-2 alleviated the need for such linkers (PDB IDs 5W5C, 5W5D).^4^

**Figure 3.**
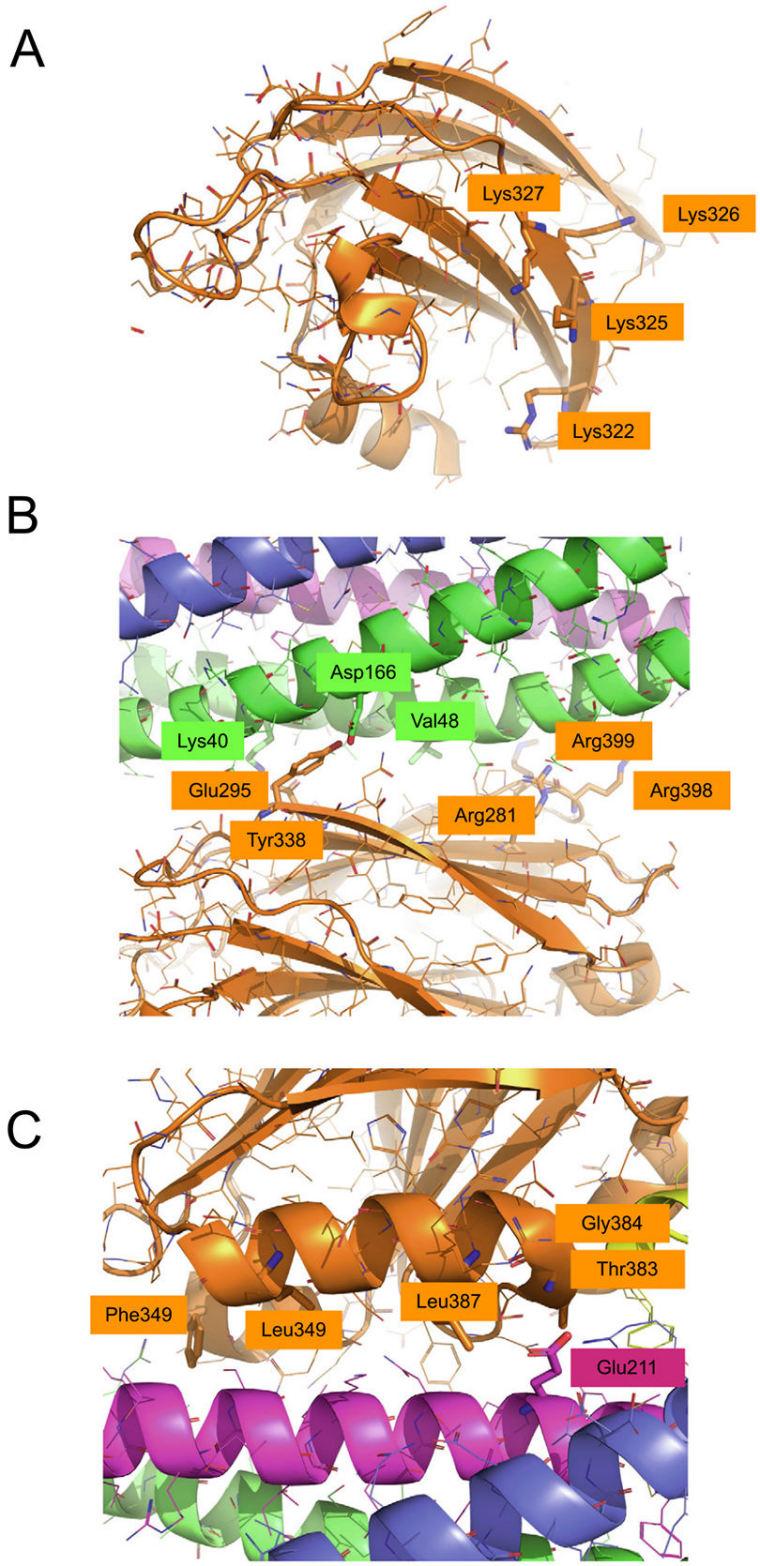
Mutations of the primary and tripartite interfaces. (A) Close-up view of the polybasic region of Syt1 C2B with residues Lys322, Lys325, Lys326, Lys327 shown as sticks (same view as in the left panel of Figure 2(A)). (B) Close-up view of the primary interface with residues SNAP-25A Lys40, Asp166, Val48 (DEE mutations) and Syt1 C2B Arg281, Glu295, Tyr338, Arg398, Arg399 (quintuple mutant) shown as sticks. (C) Close-up view of the tripartite interface with residues Syt1 C2B Thr383, Gly384, Leu387, Leu394, Phe349 and syntaxin-1A Glu211 shown as sticks. For all illustrations the crystal structure (PDB ID 5W5C) was used.

**Figure 4.**
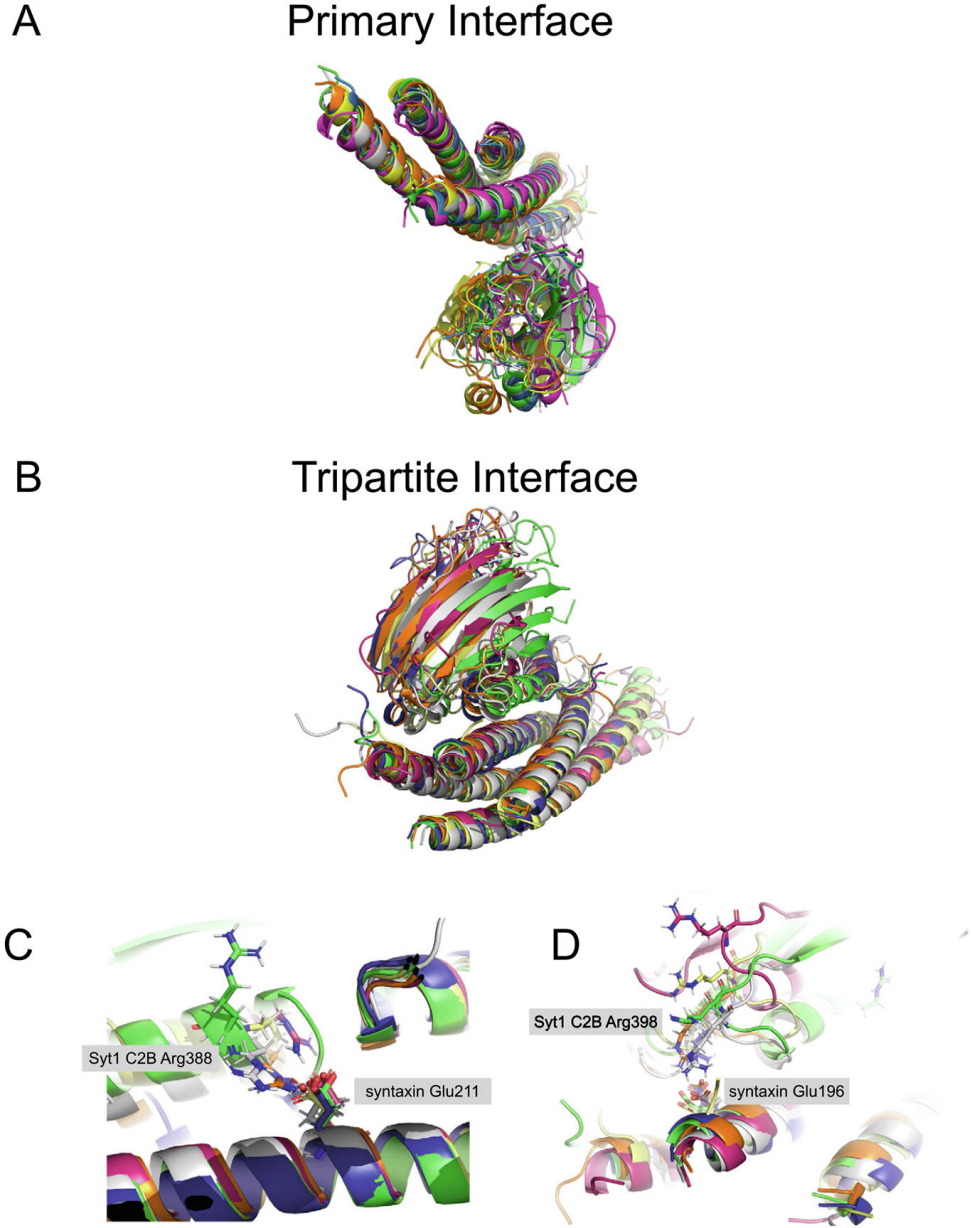
Molecular dynamics simulations of primary and tripartite interfaces. (A) End points of five independent 1-μsec simulations (colors) of the primary interface (previously published in ref. 68). Shown are cartoon representations using PyMol. Crystal structure (PDB ID 5W5C): green; simulation 1: magenta; simulation 2: yellow; simulation 3: blue; simulation 4: orange; simulation 5: gray. (B) End points of five independent 1-μsec simulations (colors) of the tripartite interface (Methods). For simulations 1 and 2, the coordinates of the molecular dynamics trajectory are shown before dissociation occurred (0.629 μsec and 0.911 μsec, respectively). Shown are cartoon representations using PyMol. Crystal structure (PDB ID 5W5C): green; simulation 1 at 0.629 μsec, magenta; simulation 2 at 0.911 μsec, yellow; simulation 3 at 1 μsec, blue; simulation 4 at 1 μsec, orange; simulation 5 at 1 μsec, gray. (C) Close-up view of a Syt1 C2B Arg388 – syntaxin-1A Glu211 predicted salt bridge that forms in 4 out of 5 simulations. (D) Close-up view of a Syt1 C2B Arg398 – syntaxin-1A Glu196 salt bridge that forms in 3 out of 5 simulations. PyMol session files corresponding to panels A and B are in Supplementary Information.

The primary interface has an interface area of approx. 720 Å^2^ that produces a complementary pattern of charge-charge interactions (Figure 2(A) and Figure 3(B)). The primary interface is not close to the Ca^2+^ binding region of Syt-1 and consequently, structures both in the absence and presence of Ca^2+^ or Mg^2+^ are very similar (PDB IDs 5CCG, 5CCH, 5CCI).^3,4^ Fully solvated molecular dynamics simulations suggest that the primary interface is energetically stable up to 1-microsecond, implying off-rates smaller than 10^−6^/sec. The five 1-microsecond simulations resulted in variations around the crystal structure without major conformational changes or dissociation events^68^ (Figure 4(A)). When the SNARE components are aligned, the all-atom root-mean-square difference (RMSD) to the crystal structure is 5 Å, with a relatively symmetric distribution around the crystal structure.

Although the primary interface has been observed in several very different crystallization conditions, this interaction is very difficult to observe in solution. This is caused by interactions between the SNARE complex and other areas of the Syt1 surface including the so-called polybasic region (Figure 3(A)). By mutation of the polybasic region (Lys322Glu/Lys325Glu) of Syt1 C2B and by using a biochemically well-behaved assembly between the SNARE complex and Cpx1, many of these other interactions can be reduced, resulting in substantial chemical shift changes around the primary interface in a ^1^H-^15^N TROSY-HSQC experiment,^6^ with an estimated K_D_ of >20 μM. Isothermal titration calorimetry (ITC) experiments with a different set of mutations of the polybasic region (Lys326Ala/Lys327Ala) suggested a K_D_ of 3.3 μM^4^, although there are some caveats with ITC experiments on this system, as discussed below. Consistent with other studies,^3^ mutation of two arginine residues involved in the primary interface (Arg398Gln/Arg399Gln, Figure 3(B)) impaired binding as assessed by ^1^H-^15^N TROSY-HSQC experiments whereas the Glu295Ala/Tyr338Trp mutation of the primary interface actually increased binding. Because the Glu295Ala/Tyr338Trp mutation disrupts Ca^2+^-triggered release in a fusion assay and in neurons,^3^ it is possible that the enhanced binding of this mutant may have caused a conformational change of the interface that disrupts function but not binding. Another possibility is that, in a model proposed by Voleti and colleagues,^6^ the mutant hinders dissociation of Syt-1 from the SNARE complex upon Ca^2+^-binding. In any case, the so-called quintuple mutant of the primary interface (Arg281Ala/Glu295Ala/Tyr338Trp/Arg398Gln/Arg399Gln, Figure 3(B)) is the strongest mutant of all tested Syt1 C2B mutants of the primary interface: It has a similar effect as that of a Syt-1 deletion.^3^

Based on the surface charge distribution of the primary complex, it was predicted to simultaneously interact with anionic membranes through the polybasic region of the Syt1 C2B domain (Figure 3(A)) and the positively charged juxtamembrane region of the SNARE complex.^3^ In the presence of anionic phospholipid membranes and absence of SNAREs, the polybasic region of Syt1 C2B primarily interacts with the membrane.^30^ Binding experiments of fluorescently labeled Syt1 C2AB with nanodiscs both with and without reconstituted SNARE–Cpx1 complexes confirmed this observation and showed that the binding affinity is greatly enhanced in the presence of the membrane reconstituted SNARE–Cpx1 complex.^6^ These findings are consistent with other binding studies.^31^ Moreover, while this study did not include full-length (membrane-bound) SNAREs, a low-resolution cryo-EM structure of the primary complex on lipid nanotubes suggests that membrane interactions with the polybasic region can co-exist with the primary interface.^69^ Molecular dynamics simulations further support the interaction of the primary-interface complex with an anionic membrane.^70^ Moreover, in these simulations, one of the residues that is critical for the primary interface (Arg 398) formed a more extensive interaction with a negative pocket formed by residues Glu55, Gln58, and Glu62 of SNAP-25A. A similar interaction is formed in five, 1-μsec molecular dynamics simulations of the primary interface alone and in the absence of membranes.^68^

The primary interface was tested with a single-vesicle fusion assay that included neuronal SNAREs, Syt1, and Cpx1. Mutations of both Syt1 C2B and SNAP-25A were designed that were expected to disrupt the primary interface based on the crystal structures. All mutations disrupted Ca^2+^-triggered fusion, but they had no detectable effects on Ca^2+^-independent (spontaneous) fusion and had varying effects on vesicle association.^3^ Physiological support for the primary interface came from experiments in neuronal cultures of conditional Syt1 knockout mice. These experiments revealed that these mutations of the C2B domain of Syt1 also disrupted evoked release, while a subset of the mutations unclamped spontaneous release. All mutations resulted in facilitation during high-frequency stimulation compared to depression for the wildtype cultures.^3^

More physiological support came from disease-related mutations of SNAP-25A that are at the primary interface (SNAP-25A Lys40Glu, Asp166Tyr, Val48Phe, Figure 3(B)). These particular mutants were uncovered by next-generation sequencing techniques of DNA from patients suffering from developmental and epileptic encephalopathies (DEEs).^71^ The three mutations were individually tested in SNAP-25 knockout mice that were rescued with wildtype or mutant SNAP-25B. The three mutations all result in substantially lowered eIPSC amplitudes and rise slopes; in other words, less Ca^2+^-triggered neurotransmitter release and less synchrony.^72^ Similar results were obtained when interface variants were overexpressed in wildtype neurons.

Additional physiological support for the primary interface came from Ca^2+^-binding site mutations that produce dominant negative phenotypes.^4,73^ A charge neutralization dominant negative mutant (aspartate to asparagine of the Ca^2+^-binding site residues) was rescued by several Syt1 C2B mutations; some localize to the primary interface in *Drosophila*.^73^ Consistent with this observation, the quintuple mutant of Syt1 C2B that disrupts the primary interface mildly rescued IPSCs, although EPSCs were not rescued using a different dominant negative mutant (aspartate to alanine) in mouse neuronal cultures.^4^ These different dominant negative effects may be related to the different type of Ca^2+^ binding site mutation (charge neutralization vs charged-to-hydrophobic) or differences in species (mouse vs *Drosophila*).

In summary, the physiological relevance of the primary interface for synaptic neurotransmitter release has been validated by a variety of studies.

### Tripartite interface

The crystal structures of the SNARE–Syt1–Cpx1 complex^4^ revealed another interface in addition to the primary interface where a second crystal-symmetry-related Syt1 molecule interacts with the SNARE–Cpx1 side of the complex (Figure 2(B)). There are two crystal forms of this tripartite interface, one with the Syt1 C2B domain at 2.5 Å resolution (PDB ID 5W5D), and another with the Syt1 C2A-C2B fragment at 1.85 Å resolution (PDB ID 5W5C). The structure of the tripartite interface is very similar in both crystal forms (r.m.s.d. = 0.30 Å), while the structure of the primary interface is very similar to that found in the SNARE–Syt1 crystal structure (PDB ID 5CCG, RMSD = 0.39 Å).

For the tripartite interface, the Syt1 C2B domain binds to the SNARE–Cpx1 subcomplex via interactions with both the SNARE and Cpx1 components (interface area 990 Å^2^). A shape and charge complementarity exists between the molecules involved in the SNARE–Cpx1–Syt1 tripartite interface (Figure 2(B)), along with hydrophobic interactions. The structure of the tripartite interface is appealing because it provides a possible explanation of the cooperation between Syt1, Cpx1, and the SNARE complex. Since the α-helix HA is structurally conserved in C2B domains of all Syts, Doc2b, and Rabphilin, but is not present in the Munc13-1 C2B, Doc2a, and Syt C2A domains, the SNARE–Cpx1–Syt1 tripartite interface may be more general and involve other Ca^2+^-binding C2 modules.

As mentioned above, the Syt1 C2B domains that are involved in the tripartite and the primary interfaces are related by symmetry in the crystal structures.^4^ This is unusual but does not necessarily suggest that one of the two interfaces is a crystallization artifact (the crystal structures are of high quality and most sidechains at the interfaces have well defined density), and it may suggest possible supramolecular arrangements.^74^ However, considering that the tripartite interface is presumably weaker than the primary interface, it may have been influenced by crystal packing. For example, the tripartite interface could represent an ensemble of conformations, and the crystallization may have favored one conformation out of this ensemble. Using the same protocols and programs that were used for the simulation of the primary interface,^68^ we tested this by performing five, 1 μsec molecular dynamics simulations of the tripartite interface (Figure 4(B)). Out of these five simulations, three simulations were stable throughout the entire simulation period of 1 μsec (Figure 4(B)), while two simulations resulted in dissociation events at 0.629 and 0.911 μsec, respectively. In contrast to the 1 microsecond molecular dynamics simulations of the primary interface (Figure 4(A)), the simulations of the tripartite interface (Figure 4(B)) move asymmetrically with respect to the crystal structure. (The all-atom RMSD to the crystals structure is 8.6 Å for the Syt1 C2B domain when the SNARE and Cpx1 components are aligned.) Taken together, the simulations suggest that the tripartite interface is weaker than the primary interface.

Two salt bridges form in the simulations of the tripartite interface: Syt C2B Arg388 – syntaxin-1A Glu211 (4 out of 5 simulations, Figure 4(C)) and Syt C2B Arg398 – syntaxin-1A Glu196 (3 out of 5 simulations, Figure 4(D)). Residue Arg398 is relatively close to SNAP-25A Glu234, Glu238, and to syntaxin-1A Arg59 via the primary interface as observed in the crystal structure (PDB ID 5W5C). Because the same Syt1 molecule forms both interfaces in the crystal structure, it is possible that crystallization led to a conformation of Arg398 that is favorable for the primary interface. Residue Arg388 forms two alternative conformations in the crystal structure, and one of these conformations could potentially form a water-molecule-mediated interaction with syntaxin-1A Glu211. In conjunction with the overall motion of the tripartite interface observed in the simulations, the Syt C2B Arg388 – syntaxin-1A Glu211 salt bridge forms.

Interestingly, there is a DEE disease-related mutation in syntaxin-1A (Glu211Lys, Glu210Lys in human)^71^ (Figure 3(C)). The wildtype Glu211 residue forms a salt bridge with Arg388 in the above-mentioned simulations of the tripartite interface. However, syntaxin-1A Glu211 also forms a salt bridge with syntaxin-1A Lys83 in the structure of the closed syntaxin-1A–Munc18-1 complex (PDB ID 3C98).^75^ Thus, the DEE mutation of Glu211 could potentially affect the closed conformation of syntaxin or the tripartite interface, or both. However, a Syt1 C2B Arg388 mutation has not yet been investigated.

To validate the tripartite interface, ITC binding experiments were performed.^4^ Two sets of Syt1 C2B mutations were designed that were expected to disrupt binding based on the crystal structure of the tripartite interface (Leu387Gln/Leu394Gln, referred to as LLQQ and Thr383Gln/Gly384Gln, referred to as TGQQ, Figure 3(C)).^4^ In the molecular dynamics simulations of the tripartite interface, these residues of Syt1 C2B generally remain in contact with the SNARE complex. In general, ITC experiments are difficult for this multi-component system and required use of mutations to reduce the effect of other interactions between Syt1 and the SNARE complex.^5,27^ Moreover, repeat ITC experiments with a different purification protocol that included a final cation exchange chromatography step for the purification of the Syt1 C2B^KA-Q^ mutant produced a smaller and opposite signal.^76^ The ion exchange step removes polyacidic contaminants, and it was originally not used for the C2B mutants with KA-Q mutations because these mutants appear in the flow-through using the conditions described in ref. 4. In contrast, the cation-exchange buffer used for the repeat ITC experiments^76^ contained 20 mM Ca^2+^, and the proteins bound to the ion exchange resin under these conditions. We note, however, that all other Syt1 C2B constructs, including wildtype C2B and the C_2_B^KA^ mutant, studied in ref. 4 included an ion exchange step, so this difference in purification protocols only applies to the ITC studies of the C2B mutants with KA-Q mutations. Thus, in retrospect, the results of the original ITC experiments for these mutants should be viewed with caution since they depend on the purification protocol.

Solution NMR measurements of relaxation effects caused by a paramagnetic probe were performed with the same samples as used in the recent repeat ITC experiments.^76^ These high sensitivity measurements did not indicate substantial populations of the tripartite interface, although they did not rule out the possibility of very low affinity binding (kD > 1 mM). Such a low affinity would be consistent with the molecular dynamics simulations of the tripartite interface that suggest relatively low stability of the tripartite interface on the 1-microsecond timescale (Figure 4(B)). It is possible that the tripartite interaction might be enhanced *in vivo* by co-localization of the proteins in the small volume around a docked synaptic vesicle or by interactions of Syt1 C2B with the membranes. (See, for example, the models suggested in Figure 3 of ref. 75). Taken together, the well-defined character of the tripartite interface in the crystal structures (PDB IDs 5W5C and 5W5D), the molecular dynamics simulations (Figure CB), and the electrophysiology suggest that the tripartite interface is a possible interface that warrants further validation.

Thus far, the strongest support for the tripartite interface comes from several electrophysiology experiments. Syt1 and its two mutants, again LLQQ and TGQQ, were separately expressed in cultured cortical neurons derived from double mutant mice harboring Syt1 conditional and Syt7 constitutive KO alleles.^4^ Both mutants are properly localized,^76^ arguing against potential folding or gross trafficking defects of these mutants.^6^ Double removal of Syt1/7 suppressed synchronous and asynchronous release and increased spontaneous mini release. These phenotypes could be fully rescued by expression of wildtype Syt1 (Syt1^WT^). The TGQQ mutant also rescued evoked IPSC amplitude and mIPSC frequency, whereas the LLQQ mutant of Syt1 failed to rescue synaptic release. These results were mirrored by experiments with a dominant negative Ca^2+^-binding mutant of Syt1 (D309A/D363A/D365A-mutant, referred to as Syt1^DA^). While Syt1^WT^ in cultured WT neurons induced no phenotype, Syt1^DA^ expression reduced the amplitudes of both evoked inhibitory postsynaptic currents IPSCs and evoked excitatory postsynaptic currents, and increased the frequencies of both miniature IPSCs and miniature EPSCs.^4^ The dominant-negative activity of Syt1^DA^ was strongly eliminated by the LLQQ mutant, whereas the TGQQ mutant of Syt1^DA^ was still dominant although slightly less strong than Syt1^DA^ itself.

The tripartite interface was further tested by experiments with Cpx1/2 double knockdown (DKD) in WT neurons. In WT neurons with exogenous Syt1^WT^ expression, the Cpx1/2 DKD partially decreased the evoked IPSC and EPSC amplitudes and increased mIPSC and mEPSC frequencies, whereas in WT neurons with exogenous Syt1^DA^ expression, the Cpx1/2 DKD partially reversed the massive dominant-negative effect of Syt1^DA^.^4^ As a result, synaptic responses in Cpx1/2 DKD neurons were identical in neurons with Syt1^WT^ and Syt1^DA^ expression. Considering the milder effect of Cpx1/2 DKD than expression of Syt1^DA^, this result cannot be explained by saturation due to overexpression. Rather, the dominant negative effect of the Syt1^DA^ mutant requires Cpx, in support of the tripartite interface since Cpx is involved in the tripartite interface. However, an alternative explanation^6^ suggested that Cpx can function in conjunction with the primary interface alone by keeping the membranes apart prior to putative dissociation of the primary interface upon Ca^2+^ binding.

Further potential physiological support for the tripartite interface comes from Syt1 C2B Phe349 (Figure 3(C)) which is involved in hydrophobic interactions with both syntaxin-1A and SNAP-25A in both the crystal structure (PDB ID 5W5C) and the molecular dynamics simulations. Mutation of Syt1 C2B Phe 349 to alanine increased spontaneous release and disrupted Ca^2+^-triggered release in PC12 cells,^77^ and increased spontaneous release, the release probability of the readily releasable pool (RRP), and the probability of synchronous release in primary cortical neuron cultures.^78^ Interestingly, studies with hippocampal Syt1 knockout cultures did not result in phenotypes with this mutant^79^; the differences between these studies are unclear. In any case, as observed in negative stain images of reconstituted Syt1 C2A-C2B fragments, this mutation also disrupts Syt1 ring formation. However, it is still uncertain if and when such rings might occur prior to formation of prefusion synaptic complexes. In summary, this phenotype of the Phe349 mutation could be explained by disruption of such rings or by disruption of the tripartite interface, or both.

### Interactions involving the polybasic region

The C2B domain contains many charged residues consisting of clusters of basic residues on the surface of the β-sandwich, including so-called polybasic region (Figure 3(A)). These regions can interact with anionic membranes both in the absence and presence of Ca^2+^, and some of these interactions are enhanced by PIP2.^6,30^ As mentioned above, some of these interactions enhance the primary interface in the absence of Ca^2+^ by simultaneous binding of basic regions of the C2B domain and the SNARE complex to an anionic membrane.^3,6^

In the absence of membranes, and in both the absence and presence of Ca^2+^, the C2B domain can also interact with the SNARE complex via another region that is distinct from both the primary and tripartite interfaces as determined by solution NMR experiments with paramagnetic relaxation enhancement (PRE) labels in the presence of a chaotropic agent.^5,23^ These experiments revealed a broad ensemble of structures where the polybasic region of the C_2_B domain binds to the acidic residues on the SNARE complex. The strong disruption of C_2_AB binding to the SNARE complex caused by mutations in the polybasic region could be consistent with this ensemble and correlated with the effects of these four mutations on neurotransmitter release in electrophysiology experiments in neuronal cultures.^5^ However, subsequent systematic studies of Syt1–SNARE and Syt1–lipid interactions using nanodiscs showed that the electrophysiological data are better explained by the effects of the mutations on Ca^2+^-dependent binding of Syt1 to PIP_2_-containing membranes.^6^ In the presence of Ca^2+^, Syt1 C2B binds to PIP_2_-containing membranes with higher affinity than to SNARE complex,^6^ suggesting that the Ca^2+^-dependent interaction in solution between the polybasic region of the Syt1 C2B domain and the SNARE complex is probably not physiologically relevant.

### Other interactions

In addition to the interactions between the SNARE complex and Syt1 C2B discussed above, there are additional possible interactions between acidic residues at the C-terminal end of the SNARE complex and the polybasic region and other regions of Syt1 C2B as observed by solution NMR experiments with PRE labels in the absence of Ca^2+^.^76,80^ These interactions cannot be explained with a single binding mode, and because each one of these regions of Syt1 contains basic residues, and the SNARE complex has abundant negatively charged regions. The relevance of these interactions is still unclear, although it is possible that upon Ca^2+^ influx, binding of Syt1 to the C- terminus of the SNARE complex releases the inhibition of neurotransmitter release caused by the complexin accessory helix^76^.

## Evidence for stable prefusion complexes

**Figure 5.**
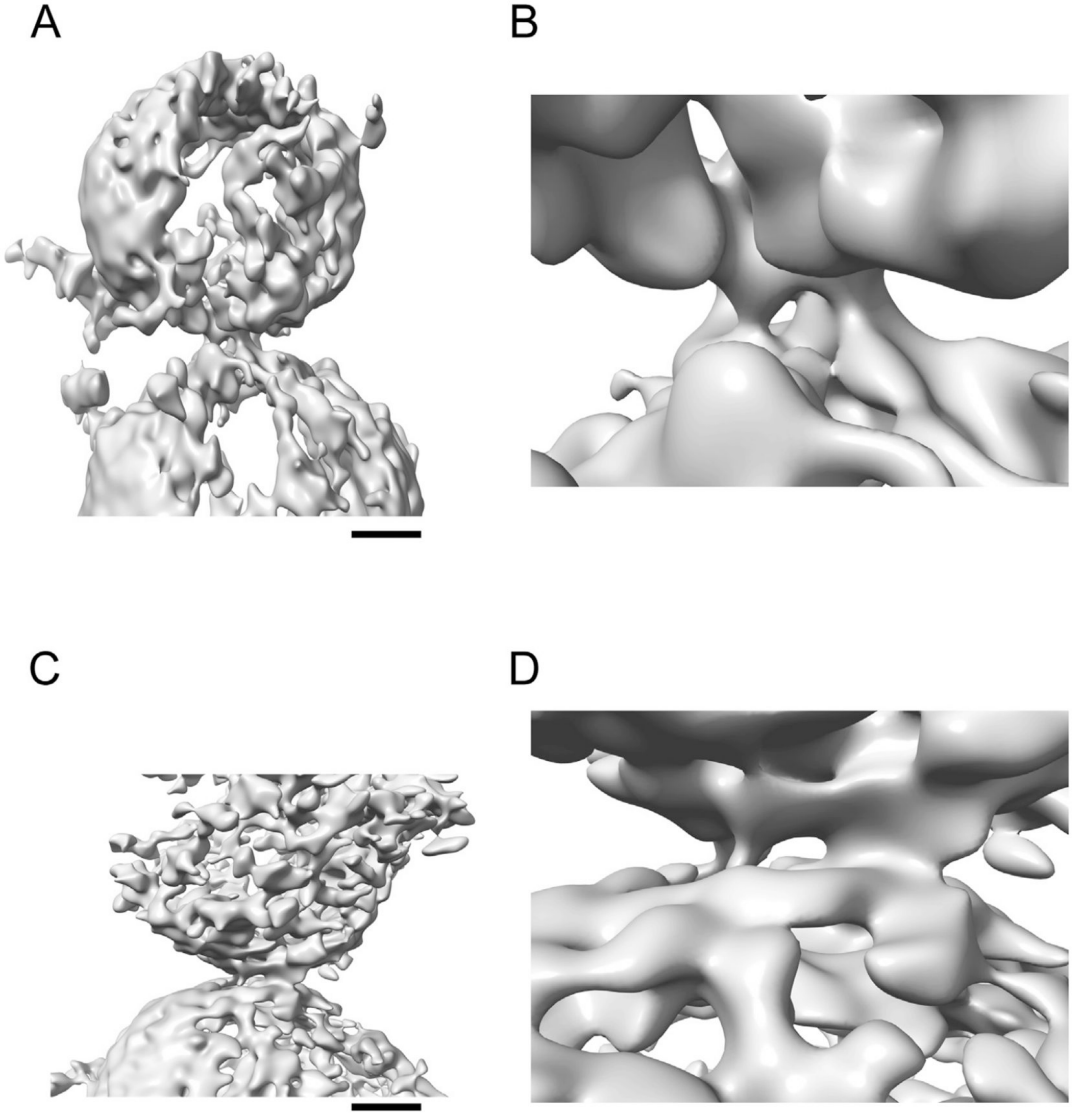
Synaptic interfacial densities. (A–D) Representative Wiener-filtered volumes^92^ of two synaptic interfaces between isolated synaptic vesicles and SM acceptor vesicles.^82^ The subtomograms were extracted with RELION with inverted contrast, a 256-pixel box size (pixel size 2.62 Å), and were normalized. Panels A, C are overview images (scale bar 10 nm), and panels B, D are corresponding close-up views.

The crystal structures of the primary and tripartite interfaces and the NMR studies of the polybasic interface between Syt1 C2 and the SNARE complex were performed in the absence of the full biological context (*i.e.*, full length proteins and membranes). Elucidating the molecular architecture of these protein complexes in a Ca^2+^-free prefusion state in the presence of membranes is an important next step to fully elucidate the mechanism of Ca^2+^-triggered exocytosis. Single vesicle fusion experiments and low resolution cryo-electron microscopy suggested the existence of stable point contacts between vesicles with reconstituted SNAREs, Syt, and Cpx in the absence of Ca^2+^,^40^ although the resolution of these EM studies was insufficient to visualize the nature of these point contacts. Nevertheless, injection of Ca^2+^ resulted in rapid fusion of most of these vesicle associations. Cryo-electron tomography studies of similar reconstituted vesicles produced first glimpses of the morphologies of these contacts in the Ca^2+^-free state and suggested that membranes are kept apart (>30 Å) by protein complexes situated between membranes.^81^ Again, addition of Ca^2+^ resulted in disappearance of most of these contacts and fused vesicles. More recently, a cryo-ET data set of synaptic protein complexes was collected in their native environment between isolated synaptic vesicles (ISVs) and synthetic vesicles that mimic the plasma membrane^82^ (Figure 5), confirming the notion that stable prefusion complexes exist between synaptic vesicles and the plasma membrane.

Subtomogram averaging is exceedingly difficult due to the relatively small size of SNAREs, Cpx1 and Syt1 (~70 kDa in total for the SNARE complex alone) in addition to the relatively low abundance of fully assembled presynaptic complexes at a given synaptic terminal. Moreover, analyses are hindered by a crowded molecular environment and the non-discrete nature of the particles (*i.e.*, they are sandwiched between extended membranes). Thus, new technologies had to be developed to analyze such cryo-ET data sets.^82^ 3D signal permutation was performed as an intervening step between subtomogram extraction using suitable masks and subsequent 3D classification and refinement. This 3D signal permutation, combined with feature-guided alignment, produced strong densities for three class average maps at the intermembrane density locations, and each of these intermembrane densities were morphologically distinct from one another. Work is in progress to collect additional tomograms to obtain higher resolution class averages.

Relatively small numbers of intermembrane densities were observed for a given pair, and there were no obvious regular arrangements between ISVs and acceptor vesicles.^82^ Typically, between 1 and 3 distinct interfacial densities were observed (Figure 5). The lack of obvious regular arrangements agrees with cryo-ET studies of synaptosomes obtained from hippocampal organotypic slice cultures from mice^83^ where a variety of tethers and contacts between docked synaptic vesicles and the plasma membranes were observed, but no regular arrangements. In contrast, apparent symmetrical arrangements were found in subtomogram averages of cryo-ET data obtained from cultured hippocampal neurons.^84^ However, the best map averages imposed sixfold symmetry and relied on top/bottom views that contain little information in the profile direction due to the missing wedge effect. Clearly, much more data are required to resolve the intermembrane densities and their arrangements.

Collectively, all cryo-ET studies support the notion of the existence of stable intermembrane complexes that are situated between synaptic vesicles and plasma membranes in the absence of Ca^2+^. As such, these prefusion complexes prevent fusion, but they bring the membranes in relatively close juxtaposition (as close as ~30 Å, Figure 5), setting the stage for triggered fusion upon Ca^2+^ binding to synaptotagmin. As suggested by recent analyses of neurotransmission of calyx of Held synapses,^85^ most of these prefusion complexes probably correspond to tightly bound synaptic vesicles.

## Inhibition of the primary interface by a competitive peptide

The primary interface involves residues on SNAP-25A and syntaxin-1A that interact with the Syt1 C2B domain. Of these interactions, a major part is the N-terminal SNAP-25A SNARE helix (~residues 37–58). One would therefore expect that a peptide fragment of SNAP-25A that includes residues from this region would act as a competitive inhibitor of the primary interface by binding to Syt1 C2B. However, this peptide would normally be unstructured, hindering its potential inhibition activity and prone to degradation. Thus, hydrocarbon staples were introduced,^86^ and a number of different designs explored.^68,87^ Several designs exhibited strong inhibition as well as a high degree of α-helicity in lipid mixing fusion assays.^87^ The one with the highest degree of a-helicity (referred to as SP9) was used for several further studies.

**Figure 6.**
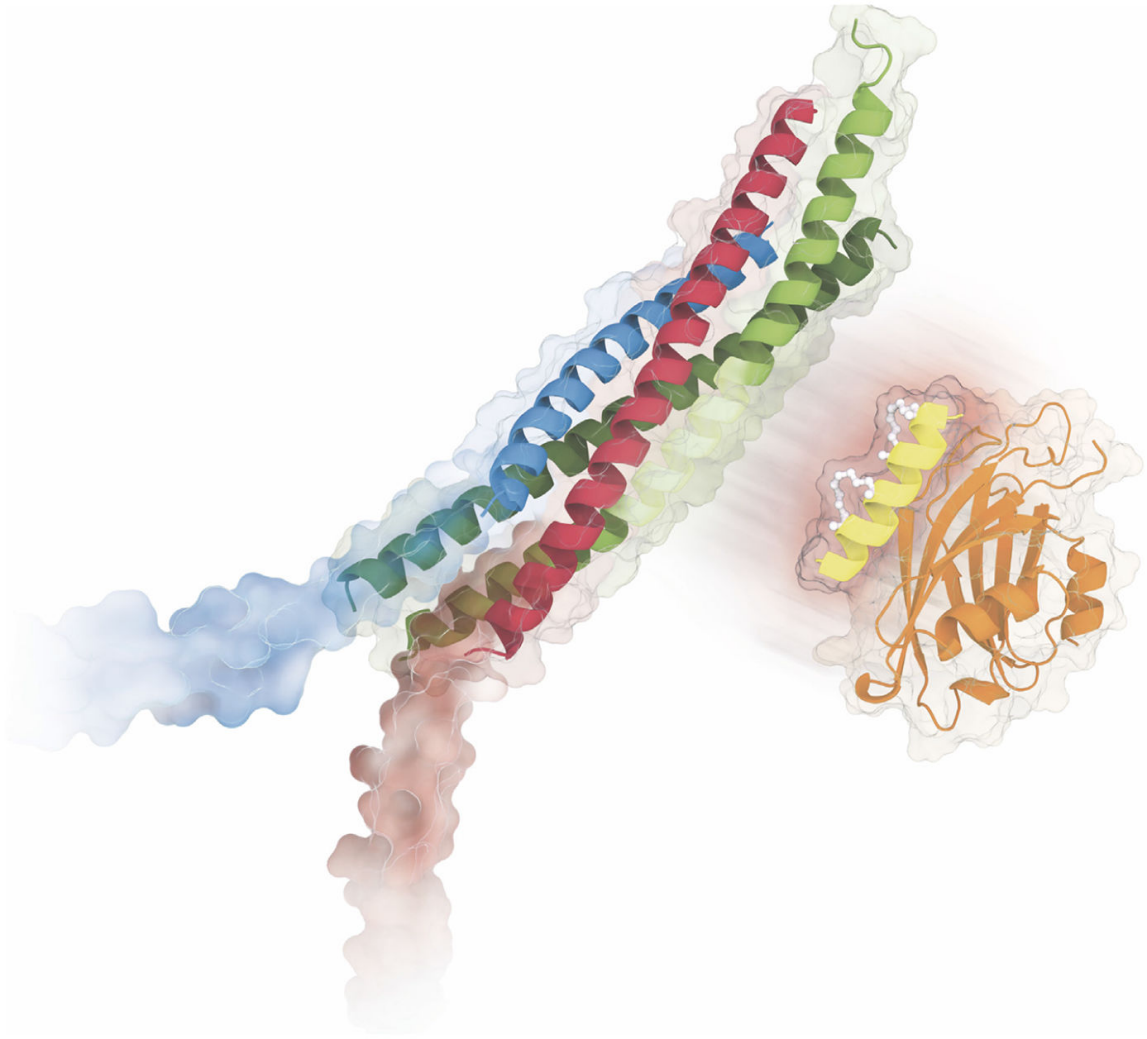
Inhibition of the primary interface. The SP9 stapled peptide (bright yellow) inhibits stimulated neurotransmitter release and mucin secretion by disrupting the interaction between Syt1 or Syt2 (orange cartoon/molecular surface) and the SNARE complex (green, red, and blue), effectively competing with the primary interface between Syt1/Syt2 and the SNARE complex. Credit: Eric D. Smith.

Based on the structure of the primary complex, SP9 is predicted to interact with Syt1 C2B, but not with the SNARE complex (Figure 6), effectively competing with the primary interface. When starting from the conformation that the peptide has in the primary interface, microsecond molecular dynamics simulations suggest that SP9 can adopt a number of binding poses that would interfere with the primary interface.^68^ In contrast, the unstapled peptide is much more variable and rapidly moves away from the starting conformation. To corroborate this notion, fluorescence anisotropy experiments between Cy3-labeled SP9 and Syt1-C2B were performed, and suggested that SP9 binds to Syt1-C2B with a K_D_ of 23 μM.^68^ In contrast, the unstapled peptide did not result in observable binding. Moreover, the “quintuple” mutant of Syt1 C2B (Arg281Ala/Glu295Ala/Tyr338Trp/Arg398Ala/Arg399Ala) that disrupts the primary interface^3^ showed only weak binding consistent with the disruption of salt bridges involving residues Arg281 and Glu295 (Figure 3(B)) which are critical for the interactions with SP9. In a single vesicle fusion experiment with reconstituted SNAREs and Syt1, SP9 resulted in a reduction of the Ca^2+^-triggered fusion amplitude by about 50%.^68^

**Figure 7.**
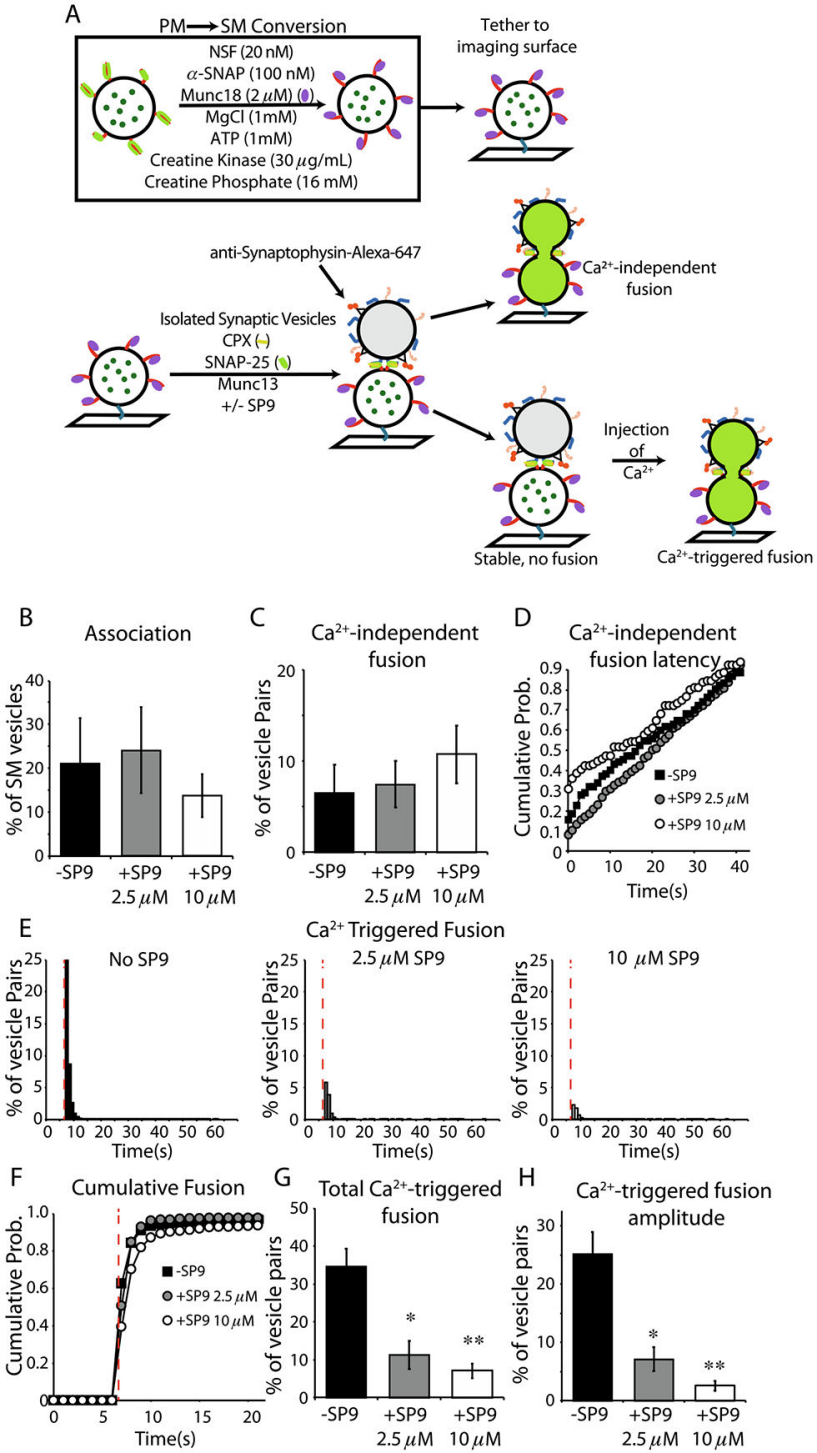
SP9 inhibits Ca^2+^-triggered fusion and synchronization with Ca^2+^ arrival but not vesicle association or stability of association. (A) Schematic workflow of the fusion assay, red lines indicate syntaxin-1A; green lines indicate SNAP-25A; purple ovals represent Munc18; blue lines represent synaptobrevin-2; orange lines represent Syt1; yellow lines present Cpx1, and green dots indicate sulforhodamine. (B) Association of SM-ISV pairs in the absence (black) or presence of 2.5 μM SP9 (gray) or 10 μM SP9 (white). (C) Ca^2+^-independent fusion rate of SM-ISV pairs. (D) Cumulative probability histogram of time between association and Ca^2+^-independent fusion of vesicle pairs in panel C. (E) Histograms of fusion time after Ca^2+^-injection (red dashed line) in the absence of SP9 (left), 2.5 μM SP9 (center), and 10 μM SP9 (right). (F) Cumulative probability distribution of the histograms in panel E. (G) Total percentage of SM-ISV pairs that fused after Ca^2+^ injection. (H) The amplitude of the first time bin after Ca^2+^ injection shows the SP9 peptide decreases the synchronization of fusion with Ca^2+^ arrival. Error bars indicate the standard error of 6 technical replicates from 3 biological replicates for No SP9 condition, 8 technical replicates from 2 biological replicates for the 2.5 μM SP9 condition, and 13 technical replicates from 3 biological replicates for the 10 μM SP9 condition.

To elucidate the effect of SP9 in more detail, we developed a more complete reconstitution using isolated glutamatergic synaptic vesicles (ISVs) from mouse brain homogenates, acceptor vesicles that contain Munc18-1–syntaxin-1A complexes, along with Cpx1, Munc13, N-ethylmaleimide-sensitive factor (NSF), αSNAP, and an ATP regeneration system (Figure 7(A)). All components were present at all stages upon addition of the ISVs to surface-tethered acceptor vesicles. The reconstitution and fusion assay are described in detail in ref. 42. Briefly, Sec1/Munc18 (“SM”) vesicles were prepared with reconstituted syntaxin-1A–Munc18-1 complexes and with encapsulated fluorescent content marker sulforhodamine. The SM vesicles were tethered to an imaging surface. ISVs were added along with Munc13-1, SNAP-25A, and Cpx1. The ISVs were labeled via a secondary fluorescent Alexa-647-conjugated synaptophysin antibody allowing co-localization of associated ISV-SM pairs. Generally, stable prefusion associations form in the absence of Ca^2+^, from which Ca^2+^-independent fusion events are relatively rare (Figure 7(B, C)). These Ca^2+^-independent fusion events have a relatively higher probability of occurring immediately (within 1 sec) upon pair association (Figure 7(D)). SP9 did not significantly alter Ca^2+^-independent fusion events (Figures 7(C, D)), suggesting that SP9 itself does not directly alter the fusogenicity of vesicle pairs.

Upon injection of Ca^2+^, Ca^2+^-triggered fusion events (at 50 μM Ca^2+^, close to the physiological range) were monitored and analyzed (Figures 7 (E-H)). In the absence of SP9, there is a rapid burst of Ca^2+^-triggered fusion events that coincides with the arrival of Ca^2+^ in the evanescent field of the TIRF microscope (Figure 7(E)). Addition of SP9 at increasing concentrations results in progressive reduction of total Ca^2+^-triggered fusion (Figure 7(G)) with a pronounced loss of synchrony as measured by the fusion amplitude (Figure 7(H)).

The primary interface between Syt1 and neuronal SNAREs is conserved among all species, and it is highly conserved among Syt1, Syt2, and Syt9, all known to be involved in fast synchronous release.^3^ Moreover, it is relatively conserved for other Ca^2+^ triggered exocytosis processes. For example, it is highly conserved for homologues involved in airway epithelial mucin hypersecretion,^68^ syntaxin-3, SNAP-23, VAMP8, and Syt-2. The residues that are at or near the primary interface are identical for Syt1/Syt2 except for Val292Cys and identical for SNAP-25A/SNAP-23 except for Lys40Glu, Leu47Ile, and Val48Thr. SP9 binds to both Syt1 C2B and Syt2 C2B with a similar dissociation constant (K_d_) of 24 μM and 35 μM, respectively.^68^ When 10 μM SP9 was added in reconstitution assay for mucin hypersecretion, the Ca^2+^-triggered fusion amplitude, the cumulative fusion probability, and the synchronization were strongly inhibited, and the continuous (as opposed to immediate) Ca^2+^-independent fusion probability was only moderately reduced compared to the Ca^2+^-triggered amplitude and cumulative fusion probabilities. Moreover, SP9 had no effect on vesicle association.

Taken together, SP9 inhibits Ca^2+^-triggered membrane fusion in reconstituted systems of neuronal exocytosis and airway mucin hypersecretion, but it does not affect spontaneous or baseline release/secretion. From a mechanistic perspective, these findings have further solidified the critical role of the conserved primary interface for Ca^2+^-triggered membrane fusion.

To enable disruption of Ca^2+^-triggered exocytosis inside cells (e.g., neurons or epithelial cells), SP9 was conjugated to cell penetrating sequences (CPPs). Peptides were applied to primary human airway epithelial cells.^68^ Conjugation of SP9 with either penetratin (PEN) or TAT CPPs resulted in substantial peptide uptake into secretory airway cells. Treatment with CPP-conjugated TAT-SP9-Cy3 or PEN-SP9-Cy3 reduced ATP-stimulated, Ca^2+^-triggered MUC5AC. Importantly, baseline secretion was not affected by any of the peptides in the IL-13 (metaplastic) cultures, consistent with the absence of an effect of Syt2 deletion on baseline secretion in mice.^88^ The efficacy of CPP-conjugated SP9 was also tested in mice.^68^ Short-term treatment of mice with aerosolized PEN-SP9-Cy3 resulted in substantial peptide uptake into distal airway epithelial cells, and reduced methacholine-stimulated, Ca^2+^-triggered mucin secretion and airway mucus occlusion, whereas the non-stapled PEN-P9-Cy3 peptide did not exhibit an inhibitory effect. This peptide is expected to be a lead towards a new therapeutic to combat acute exacerbation of airway disease (asthma, chronic obstructive pulmonary disease, and cystic fibrosis).

## Concluding remarks

Recent atomic resolution structures (Figure 2), low-resolution imaging of synaptic prefusion complexes in situ (Figure 5), and functional studies have established that stable prefusion complexes juxtapose synaptic vesicle membranes close (up to ~30 A) to the plasma membrane. Multiple such prefusion complexes can exist for a particular docked synaptic vesicle, although the precise supramolecular arrangements remain to be established. In any case, these prefusion complexes prevent membrane fusion even though membranes are relatively closely juxtaposed. Upon Ca^2+^-binding to the C2 domains of Syts, the inhibition is released, and fusion is triggered on a fast time scale, most likely by the action of SNAREs and possibly also by an active role of Syts upon Ca^2+^ binding. However, the details of these processes that occur upon Ca^2+^-triggering remain to be uncovered.

## Methods

### Fusion experiments with isolated synaptic vesicles

The fusion assay has been described previously.^41,42^ Briefly, synthetic plasma membrane mimic (PM) vesicles containing the self-quenching dye, sulforhodamine B, reconstituted syntaxin-1A, and SNAP-25A were incubated in an “SM conversion” solution containing, 20 nM NSF, 50 nM α-SNAP, 2 μM Munc18, 1 mM MgCl, 1 mM ATP, 5 U/mL Creatine Kinase, and 15 U/mL Creatine phosphate to convert the starting complex from syntaxin-1A–SNAP-25A to syntaxin-1A–Munc18-1 (SM) containing vesicles. These SM vesicles are then tethered to the imaging surface of custom-made microfluidic slides via biotin-peg-neutravidin-biotin-PE interactions. Isolated synaptic vesicles (ISVs) were purified from whole mouse brain homogenate, similar to previous methods^89^; however in contrast to previous isolations, vesicles here were gently competitively eluted using a small peptide corresponding to the antibody epitope. ISVs were then incubated in 1:1000 anti-synaptophysin-Alexa645 overnight followed by 2hr dialysis in a 300KD-cutoff dialysis cassette to remove unbound antibody. We then added SM conversion mixture proteins to the same final concentration as above and additionally included soluble accessory proteins with final concentrations of 500 nM C1C2BMUNC2C, 1 mM SNAP-25A, and 1 mM Cpx1.

SM vesicles were used to find an appropriate imaging area, after which ISVs are added to the imaging chamber, and vesicle-vesicle association is monitored for 1 minute. Following association, the chamber was washed with 40 column volumes (200 mL) of buffer containing the SM conversion components as well as C1C2BMUNC_2_C, SNAP-25A, and Cpx1. After wash, imaging was resumed, and after 30 frames (6 seconds), we injected the same buffer solution supplemented with 50 mM Ca^2+^ and free Alexa647 dye to track Ca^2+^ arrival. Fusion assay results were analyzed with a Matlab using custom scripts (available in the Zenodo repository https://zenodo.org/record/7159049.) Imaging data are deposited in the Dryad repository https://doi.org/10.5061/dryad.280gb5mss.

### Molecular dynamics simulations

The molecular dynamics simulations of the tripartite interface described in this work (Figure 4(B)) were carried out using the same protocols and procedures for the molecular dynamics simulations of the primary interface as described in ref. 68 and shown in Figure 4(A). At variance to the previously published primary interface simulations, Cpx1 was included in the simulations of the tripartite interface. Specifically, the following residues were included in the simulations of the tripartite interface: Syt1 C2B [270–419], synaptobrevin-2,Stx1A[191–244], SNAP-25A[10–74 & 141–194], and Cpx1. For these simulations, the starting models were placed in a 111 × 111 × 111 Å periodic boundary condition box. The empty space in the box was filled with 41,204 water molecules using the VMD solvate plugin. The system has a total of 130,133 atoms. The system was charge-neutralized and ionized by addition of 129 potassium and 112 chloride ions, corresponding to a salt concentration of ~145 mM. Identical to the primary interface simulations, the CHARMM36 all-hydrogen force fields and parameters^90^ were used with a non-bonded cutoff of 11 Å. Five independent 1-μsec simulations were performed using different initial random number seeds. Trajectories of the molecular dynamics simulations of the tripartite interface are deposited in the Dryad repository https://doi.org/10.5061/dryad.f1vhhmh15.

## Supplementary Material

PyMol session file for MD simulation of the primary interface

PyMol session file for MD simulation of the tripartite interface
